# Identity and Reproductive Aspects in Females with Fragile X Syndrome

**DOI:** 10.1089/whr.2021.0059

**Published:** 2021-11-02

**Authors:** Sarah Reiss, Laura Zalles, Catherine Gbekie, Reymundo Lozano

**Affiliations:** ^1^ Department of Genetics and Genomic Sciences, Icahn School of Medicine at Mount Sinai, New York, New York, USA.; ^2^ Department of Psychiatry, Icahn School of Medicine at Mount Sinai, New York, New York, USA.; ^3^ Department of Pediatrics, Icahn School of Medicine at Mount Sinai, New York, New York, USA.

**Keywords:** Fragile X syndrome, FXS, *FMR1* gene, psychosocial, genetic counseling, reproductive decision-making

## Abstract

***Purpose:*** Fragile X Syndrome (FXS) is caused by a full mutation in the FMR1 gene, defined by >200 CGG repeats. It is the leading cause of inherited intellectual disability, but presents with a wide range of clinical variability in males and particularly amongst females. This article aims to review the perspectives of women with the full mutation in relation to Fragile X Syndrome identification, romantic desires, and reproductive decision making.

***Methods:*** We generated an online survey of 33 questions to be administered to 31 women that had visited our Fragile X Syndrome Clinic and members of the National Fragile X Foundation. We extrapolated common themes from the obtained data.

***Results:*** The results showed that most women often struggled with identifying as a female with FXS. Furthermore, many women are interested in childbearing, however most are in need of genetic counseling.

***Conclusions:*** Further research to advance the understanding of the specific needs of women with FXS is necessary.

## Introduction

Fragile X syndrome is a trinucleotide repeat expansion disorder of more than 200 CGG nucleotides in the 5′ untranslated region (5′UTR) of the Fragile X mental retardation 1 (*FMR1)* gene, which is located on the X chromosome. The expansion in this gene silences the expression of Fragile X mental retardation protein (FMRP) leading to decreased neural plasticity and abnormal synaptic function, explaining the neurological deficits and clinical manifestations that accompany Fragile X syndrome (FXS). These manifestations can include moderate to severe intellectual disability, behavioral problems, and a high incidence of autism.^1^ As the *FMR1* gene resides on the X chromosome, males present with a more severe phenotype. This is due, in part, to a naturally occurring process known as X-inactivation in females. During X-inactivation, one copy of the X chromosome randomly inactivates during development to “compensate” for the second X chromosome in females.^2^

The phenotype of females with FXS is extremely variable, and prognosis often does not come to the attention of medical professionals unless a diagnosis of FXS is made in a male family member. Although phenotypes are inconsistent from patient to patient, there is still a moderate prevalence of common characteristics. About 25% of females with FXS have an intelligence quotient (IQ) below 70, and another 25% have an IQ in the borderline range of 70 to 85.^3^ About one-third to one-half of females have normal intellectual functions.^4^ However, even in females with normal IQs, learning disabilities, executive function deficits, and attention deficits may occur.^1^ Difficulties with math are particularly common, which often makes managing money difficult, even for those who live more independently.^5^ Due to the fact that most females with FXS have normal or borderline IQ scores, many achieve higher levels of education, hold jobs, and live independently.^6^ However, emotional and psychiatric problems can be common, including social anxiety, depression, self-injury, excessive shyness, withdrawal, poor eye contact, and phobias.^7,8^ Some of these emotional and behavioral problems can be debilitating, limiting independence and depreciating quality of living.

In summary, females with the FXS full mutation often present with a milder and more variable phenotype than their male counterparts and are less frequently studied than men because their deficits are evidently less severe. To date, there are no clinical criteria that qualify a diagnosis of FXS in females. The focus of this study is to better understand the clinical and psychosocial needs of females with full mutations and how they relate FXS to their identities and making reproductive decisions.

## Materials and Methods

Eligible participants were females older than the age of 18 years with a diagnosis of Fragile X syndrome full mutation (>200 CGG repeats). Reading and writing capabilities were required to self-report on an anonymous online 33-question survey (Full survey available in Supplementary Data). The survey was piloted in a small group of subjects and professionals (pediatricians, geneticists, and genetic counselors). The language of the questionnaire was adapted for a seventh-grade level. Participants were recruited through the Mount Sinai Fragile X Clinic and the National Fragile X Foundation website. Data analysis was completed utilizing SPSS Statistics Software Version 22 and included frequencies, descriptive statistics, and correlation analyses. The open-ended responses were read and organized based on common themes. This study was approved by the Icahn School of Medicine at Mount Sinai Institutional Review Board.

## Results

### Demographic data

A total of 57 individuals responded to the survey. Of those, 31 patients responded to all the questions. Of those 31 patients, 25 reported that they have full mutations, 4 reported both premutation and full mutation, and 2 were unsure. Demographic information was collected and is displayed in Table 1. The majority of the respondents identified as white (28/90%) and were between the ages of 25 and 44 years (19/61%). The highest level of education of 13 (42%) respondents was graduating high school, while 18 (58%) subjects achieved advanced degrees. Finally, about half (14/47%) of the respondents were single and never married.

**Table 1.** Survey Respondent Demographics

	Total (*n*)	%
Age (years)		
18–24	3	10
25–34	10	32
35–44	9	29
45–54	6	19
55+	3	10
Ethnicity/Race		
White	28	90
Hispanic or Latino	2	7
Black or African American	1	3
Highest level of education		
High school diploma or equivalent	13	42
Associate degree	4	13
College degree	10	32
Postgraduate degree	4	13
Marital status		
Single, never married	14	47
Married	11	37
Divorced	4	13
Widowed	1	3
Learning disabilities		
Yes, mild	12	39
Yes, moderate	8	26
Yes, severe	2	6
No	9	29
Living accommodations		
Coreside with parents or family (nonspouse)	16	52
Independently	15	48
Group home	0	0
Employment		
Not working	9	29
Part time	9	29
Full time	13	42
Assistance with activities of daily living		
No assistance	25	81
Minimal assistance	4	13
Moderate/considerate assistance	2	6
Medical comorbidities		
Autism spectrum disorder	3	10
Aggression	2	6
Anxiety	10	32
Depression	13	42
Dyslexia	1	3
Hyperactivity	1	3
Inattention	5	16
Sensory problems	6	19
None of the above	5	16

### Fragile X syndrome-related data

Of the total respondents, 9 (29%) reported no learning disabilities, 12 (39%) reported mild learning disabilities, and 10 (32%) reported moderate or severe learning disabilities. About half of the participants lived at home with family members, while the other half lived independently. When asked about employment, 9 (29%) reported that they are not working, 9 (29%) reported that they are working part time, and 13 (42%) reported that they are working full time (Table 1). Of the 22 individuals who reported that they are employed, the most common reported occupational fields were education, food service or cleaning, nonprofit work, and administrative support. Other fields included law, photography, cosmetology, health care, government, and sales/retail.

Most of the respondents (25/81%) did not require assistance with activities of daily living. Of the 6 (19%) who required assistance, 4 (13%) required minimal assistance and 2 (6%) required moderate or considerate levels of assistance. This assistance included guidance in cooking, medical care, finances, and driving. There were no reports of requiring assistance in dressing, personal hygiene, toileting, or mobility.

In addition, the majority of the participants (26/84%) had at least one medical comorbidity; the most common diagnoses included depression (13/42%) and anxiety (10/32%). Further reported comorbidities included 3 (10%) subjects with autism spectrum disorder, 5 (16%) with inattention difficulties, and 6 (19%) with sensory processing difficulties (Table 1).

### Identity of Fragile X syndrome

**FIG. 1.**
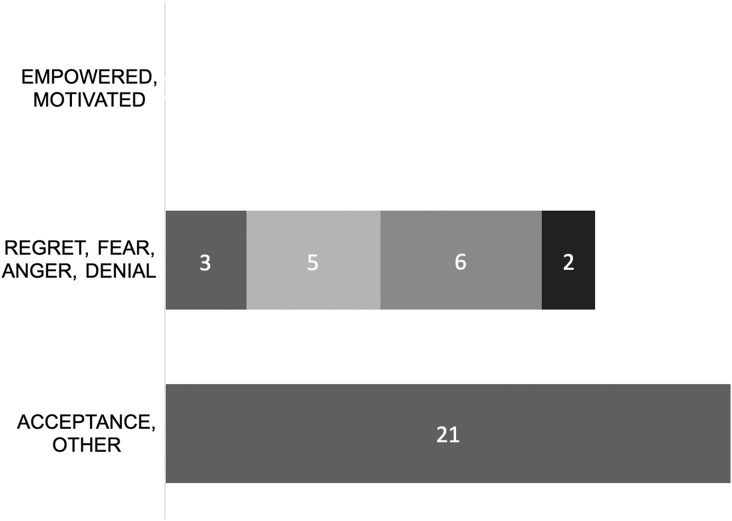
Responses to question 15: “How would you describe the way you relate to your diagnosis of Fragile X syndrome? (Please select all that apply).”

About 55% of survey responders learned that they had FXS in adulthood, with most not learning of their diagnosis until older than 30 years of age. Fourteen (45%) subjects learned that they had FXS in infancy, childhood, or adolescence (Table 2). Participants were also asked to select descriptors relating to how they feel about having Fragile X syndrome. Twenty-one (67%) of responses indicated feelings of acceptance, and of those, 3 (10%) also had feelings of anger or fear to the FXS diagnosis. There were no reports of feeling empowered or motivated, which were two of the optional positive descriptors. Respondents also had the option of selecting negative feelings; 6 (20%) described feeling angry, 5 (16.7%) were fearful, 3 (10%) had feelings of regret, and 2 (6.5%) felt that they were in denial (Fig. 1). We then asked participants to explain these feelings toward their identity.

**Table 2.** Responses to Questions Related to Fragile X Syndrome Diagnosis, Sexuality, and Reproductive Decision-Making

	Total (*n*)	%
Around what age do you remember learning that you had Fragile X syndrome?		
I have always known I had FXS	1	3
Childhood (5–10 years)	7	23
Adolescents (11–19 years)	6	19
Young adulthood (20–29 years)	3	10
Adulthood (30+ years)	14	45
Do you feel comfortable being seen by medical professionals (*e.g.*, doctors, nurses, genetic counselors) for Fragile X syndrome?		
Yes, I feel comfortable being seen for FXS by medical professionals	17	63
Somewhat, I go to appointments because they are important for my health, but I do not like being seen for FXS	4	15
No, I feel misunderstood	6	22
Are you currently in a relationship?		
Yes	16	53
No	14	47
If no, do you wish to be in a relationship?		
Yes	5	36
No	4	28
Sometimes	5	36
Do you currently have any children?		
Yes	15	50
No	15	50
If you do not have children, do you wish to have children in the future?		
Yes, I want to have children	6	40
I have wanted to have children in the past, but I have changed my mind	1	7
I am undecided	2	13
No, I do not wish to have children	5	33
I do not think I am capable of having children	1	7

FXS, Fragile X syndrome.

A variety of responses were submitted ranging from positive feelings of hope, relief, surprise, and acceptance to negative feelings of hatred, denial, isolation, and being different.

Responses with positive perspectives included the following: “At first I was very afraid of what my future would hold but after meeting with a counselor I realized there is hope for me”; “Initially, I was genuinely surprised that I had the full mutation because I have no symptoms. I was also relieved because it means that I don't have to be concerned with FXTAS”; “Learning why I behave a certain way in situations…”

Responses with negative perspectives included “I don't think I am any different than anybody else, but apparently I am. I never had any friends. Was not included in social groups. Could not keep up with other kids in sports, dance, clubs, etc.”; “My children also have FXS and I don't like it”; “I know I have it but I act like I don't know it affects me…”

Participants were then asked to select which label of their diagnosis they identify with; they were instructed to select all options that applied. Only 21% of respondents selected to identify as a female with Fragile X syndrome. The majority of those who preferred a different diagnosis identified as full mutation females, 14% of respondents identified as an affected female, 12% identified as an unaffected female, and 17% of individuals selected that they do not like being labeled as someone with FXS. Two individuals elected to write in their own responses: “just me” and “female with a full mutation or female with Fragile X syndrome.”

Next, participants were asked to report the level of comfort they feel toward sharing their diagnosis with others. Of note, four participants did not respond to this question. The majority of participants (23/77%) who did respond reported feeling very comfortable, somewhat comfortable, or only comfortable with sharing their diagnosis with close friends or relatives. Six (20%) reported that they are somewhat or very uncomfortable with sharing their diagnosis. Participants were also asked about the level of comfort they feel when being seen by medical professionals; 17 (63%) reported that they were very comfortable being seen by a medical professional for their diagnosis and 10 (37%) reported that they felt some level of discomfort. Six (22%) reported that they felt misunderstood when seen by medical professionals and 4 (15%) reported that they are seen by medical professionals because they feel it is important for their health, however, they do not enjoy being seen for FXS (Table 2).

### Reproductive decision-making

Participants were then asked a series of questions related to reproductive decision-making. Sixteen (53%) of respondents were currently in a relationship, while 14 (47%) of respondents were not. Out of the 14 individuals not in a relationship, 10 (72%) indicated that they did or sometimes wished they were in a relationship. The population was split exactly when asked if they had children or not. Out of the 15 individuals who had children, 12 (80%) respondents were pregnant before learning about their diagnosis. Out of the 15 individuals who did not have children, 6 (40%) indicated that they did want children, 1 participant reported that she previously wanted children, but has since changed her mind, 2 (13%) were undecided, 5 (33%) did not wish to have children, and 1 participant did not think she was capable of having children (Table 2).

**FIG. 2.**
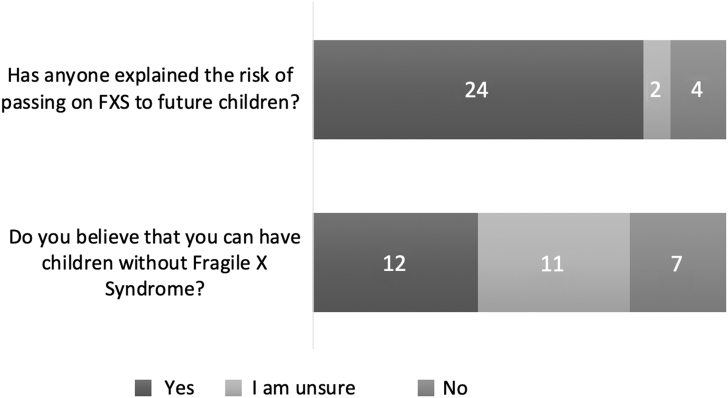
Responses to questions regarding reproductive decision-making.

Participants were then asked questions related to passing on Fragile X syndrome to a future child. First, they were asked if anyone has ever explained their risk of having a child with FXS; 24 reported yes (84%), 4 reported no (13%), and 2 indicated that they were unsure (7%). When asked if they believed they could have a child without FXS, 12 (40%) of respondents indicated yes, 7 (23%) responded no, and 11 (37%) were unsure (Fig. 2).

Finally, subjects were asked about how they feel about having a child with Fragile X syndrome. The majority of participants (16/55%) indicated that they would feel conflicted or would not want a child with FXS, and a few (3/10%) of participants felt that they would feel more connected with the child. Six (21%) of respondents selected “other,” and most of these women wrote that they were unable to answer this question because they already had children with Fragile X syndrome. When asked whether or not they would opt to use *in vitro* fertilization (IVF) with preimplantation genetic diagnosis (PGD) to decrease the risk of having children with FXS, 20 (74%) of women who responded reported that they would either definitely or potentially consider using IVF with PGD. Seven (26%) women indicated that they would not use this option, 4 (15%) for mostly moral reasons, 1 (4%) for mostly economic reasons, and 2 (7%) would prefer adoption to IVF with PGD.

## Discussion

This is the first study to explore the identity and reproductive aspects in females with Fragile X syndrome. In this study, a wide range of clinical severity was captured. The majority of respondents reported having mild learning disability and had at least one co-occurring medical condition, including depression, anxiety, sensory problems, inattention, and/or autism spectrum disorder. This study is representative of a more independent FXS population with about half of participants living and operating independently. Further, more than half of respondents had achieved an advanced degree, and the majority did not require assistance with activities of daily living.

When asked about identifying with their diagnosis, most of the participating women did not identify with the diagnosis of FXS. Many of the women who did not identify with the diagnosis of FXS achieved high levels of independence and did not have co-occurring medical conditions. Of those who did report co-occurring medical conditions, the perception of what role Fragile X syndrome played in their symptoms varied, with both positive and negative feelings about their diagnosis noted in the qualitative comments. Most of the participants felt either uncomfortable or somewhat comfortable sharing their diagnosis with professionals and others. This is an important consideration because females with the full mutation may not seek medical advice and may isolate from their community or family members. It is important to note that there was a commonality among all identity diagnoses: feeling abnormal, out of place, and misunderstood. More than a third of women reported that they struggled with their diagnosis in some way. A diagnosis of FXS may be further complicated for females because of their placement in the same genetic category as men, despite the fact that they present with different phenotypes. It is important for health care practitioners of all specialties that care for women to recognize the challenges that some women with Fragile X syndrome full mutation face.

Previous efforts have been made to use more inclusive terminology, including “Fragile X Spectrum Disorders”^3^ and “Fragile X-Associated Neuropsychiatric Disorders,” which were introduced to include disorders not related to intellectual disability such as anxiety, depression, attention-deficit/hyperactivity disorder, and obsessive-compulsive disorder in Fragile X premutation carriers.^9^ To date, there are no clinical criteria for the diagnosis of FXS in females, and the diagnosis is solely based on molecular findings.

In this study, it was found that the majority of women were interested in having children—one half already had children, and of those who did not have children, wanted children in the future. A majority of women were either conflicted about or did not want to have a child with FXS, and the majority of women would use or would consider using IVF with PGD to prevent having a child with FXS. Of the women who had children, most of them were pregnant before receiving their FXS diagnosis. This is important because, as aforementioned, the majority of women with FXS would rather not have a child with FXS if they had the option. Remarkably, about half of participants knew that they were capable of having a child without FXS. This exhibits a gap in knowledge among this population, and the need for more effective counseling and consideration of a referral to a reproductive specialist upon diagnosis.

The recommendations detailed by the National Society of Genetic Counselors noted that “intellectual disability, concreteness, inconsistent attitudes, and tangential thinking may limit the success of traditional genetic counseling methods which emphasize medical education when counseling a female with full mutation.” It is recommended that these women may benefit from an exploration of their feelings rather than the traditional education-based model.^10^

Limitations of this study arise from the study design. Providing an online survey may have produced a biased population which included a higher functioning or more independent respondent group requiring skills in reading comprehension, navigating technology, and maintaining attention.

## Conclusion

Fragile X syndrome presents with a more severe phenotype in male patients. However, about half of the women with Fragile X syndrome are still affected and must learn how to manage their symptoms and diagnosis. When asked about identifying with their diagnosis, most of the participating women identified with a label different than FXS. Women felt uncomfortable sharing their diagnosis with others, discomfort being seen by medical professionals for FXS, and misunderstood by professionals. The majority of women were either conflicted or did not want to have a child with FXS, and most women would consider or pursue using IVF with PGD to prevent having a child with FXS. Future research should be conducted to better describe the phenotype of females with FXS and how they receive, accept, and identify with their diagnosis as well as how to best serve them with genetic counseling.

## Supplementary Material

Supplemental data

## Abbreviations Used

5′UTR: 5′ untranslated region
FMR1: Fragile X mental retardation 1
FMRP: Fragile X mental retardation protein
FXS: Fragile X syndrome
IQ: intelligence quotient
IVF: *in vitro* fertilization
PGD: preimplantation genetic diagnosis
