# Supplementary material for: Identity and Reproductive Aspects in Females with Fragile X Syndrome
Source: Womens Health Rep (New Rochelle). 2021 Nov 2;2(1):500–6. doi: 10.1089/whr.2021.0059 (PMC8617583; doi:10.1089/whr.2021.0059)
Supplement: Supplemental data [file Suppl_Data.docx]

**Supplemental Information:**

Research Information Sheet

The purpose of this research study is to understand the perspectives of women with Fragile X Syndrome on how they identify with their diagnosis and make reproductive decisions. You are being asked to take part in a research study because you are a female and have a full Fragile X mutation.

Being in a research study is completely voluntary. You can choose not to be in this research study. You can also say yes now and change your mind later. Deciding not to be in the research study, now or later, will not affect your ability to receive medical care at Mount Sinai Medical Center or your membership at the National Fragile X Foundation.

If you agree to take part in this research, you will be asked to complete the attached questionnaire. Your participation in this study will take about 5-10 minutes. We expect that about 30 people will take part in this research study.

You can choose not to answer any question you do not wish to answer. You can also choose to stop taking the survey at any time. You must be at least 18 years old to participate. If you are younger than 18 years old, please stop now.

The possible risks to you in taking part in this research are:

- Feeling uncomfortable when answering questions or having someone else find out that you were in a research study.

To protect your identity as a research subject, no identifiable information will be collected. In any publication about this research, your name or other private information will not be used.

If you have any questions about this research, please contact the Researcher at Sarah Reiss at 212-241-6947. You can also call the Program for the Protection of Human Subjects Office at 212-824-8200.

**Study Survey**

Part 1: Demographics

1. What is your gender?
   1. Female
   2. Male
   3. Other (Please Specify)
2. What is your age?
   1. 18-24 years old
   2. 25-34 years old
   3. 35-44 years old
   4. 45-54 years old
   5. 55 or older
3. What is your ethnic origin or race? (Please select all that apply)
   1. White
   2. Hispanic or Latino
   3. Asian or Pacific Islander
   4. Black or African American
   5. Other
4. What is the highest degree or level of education you have completed?
   1. High School diploma or equivalent
   2. Associate Degree (e.g. AA, AS)
   3. College Degree (e.g. BS, BS)
   4. Post Graduate Degree (e.g. MA, PhD)
5. What is your marital status?
   1. Single, never married
   2. Married
   3. Divorced
   4. Separated
   5. Widowed

Part 2: Medical/Psychosocial Concerns

1. Around what age do you remember learning that you had Fragile X Syndrome?
   1. I have always known that I had Fragile X Syndrome
   2. Childhood (5-10 years)
   3. Adolescence (11- 19 years)
   4. Young adulthood (20-29 years)
   5. Adulthood (30+ years)
2. Do you have learning disabilities?
   1. Yes, severe
   2. Yes, moderate
   3. Yes, mild
   4. No
3. Do you have any of the following? (Please select all that apply)
   1. Aggression
   2. Anxiety
   3. Autism Spectrum Disorder
   4. Dyslexia
   5. Depression
   6. Hyperactivity
   7. Inattention
   8. Self-injury
   9. Sensory Problems
   10. None of the Above
   11. Other
4. Where do you currently live?
   1. Co-reside with parents or other family members
   2. Group home
   3. Independently
   4. Other
5. Are you currently employed?
   1. Not working
   2. Part time
   3. Full time
6. If yes to Question #10, which of the following best describes your job?
   1. Education
   2. Arts/design/entertainment
   3. Business
   4. Healthcare/ Research
   5. Working with children
   6. Office and Administrative Support
   7. Working with individuals with disabilities
   8. Law
   9. Sales/retail
   10. Other
7. On a scale of 1-5 with 5=the fastest fast and 1= the slowest, how fast paced is the environment in which you work?
   1. 1
   2. 2
   3. 3
   4. 4
   5. 5
8. Do you need assistance in activities of daily living?
   1. No assistance
   2. Minimal assistance
   3. Moderate/considerate assistance
9. Please explain the level of assistance required.
10. How would you describe the way you relate to your diagnosis of Fragile X Syndrome? (Please select all that apply)
    1. Acceptance
    2. Anger
    3. Denial
    4. Empowered
    5. Fear
    6. Motivated
    7. Regret
    8. Other
11. Did you always feel this way?
    1. Yes
    2. No

Please explain any previous feelings towards your identity or any adjustment period you went through:

1. With which of the following “labels” of your diagnosis would you identify? Select all that apply.
   1. Fragile X Female
   2. Full Mutation Female
   3. Affected Female
   4. Unaffected Female
   5. I do not like being labeled as someone with Fragile X Syndrome
   6. Other
2. Do you feel comfortable sharing your diagnosis with other people?
   1. Very comfortable
   2. Only with close friends or relatives
   3. Somewhat comfortable
   4. Neutral
   5. Somewhat uncomfortable
   6. Very uncomfortable
3. Do you know any males with Fragile X Syndrome?
   1. Yes, close friends/family
   2. Yes, acquaintances
   3. No
4. Do you feel as though they have impacted your view of Fragile X syndrome? Please explain.
5. Do you feel comfortable being seen by medical professionals for Fragile X Syndrome (e.g. doctors, nurses, genetic counselors?)
   1. Yes, I feel comfortable being seen for Fragile X Syndrome by medical professionals.
   2. Somewhat, I go to medical appointments because they are important for my health, but I do not like being treated for Fragile X Syndrome.
   3. No, I feel misunderstood by medical professionals, and I do not want to attend these appointments. Please explain:

Part 3: Sexuality/ Reproductive Decision Making

1. Are you currently in a relationship?
   1. Yes
   2. No
2. Do you wish to be in a relationship?
   1. Yes
   2. Sometimes
   3. No
3. Are you concerned about being taken advantage of in a relationship?
   1. Yes
   2. No
   3. I am unsure
4. Do you currently have any children?
   1. Yes
   2. No
5. If you do have children, did you have them before or after your diagnosis of Fragile X Syndrome?
   1. Before my diagnosis
   2. After my diagnosis
   3. Other
6. Do you want to have children (or more children) in your future?
   1. Yes, I want to have children.
   2. I have wanted to in the past, but I have changed my mind.
   3. I am undecided.
   4. I do not think I am capable of having children.
   5. No, I do not wish to have children.
7. Do you believe you can have children without Fragile X Syndrome?
   1. Yes
   2. No
   3. I am unsure
8. Has anyone explained the risk of passing on Fragile X Syndrome on to future children?
   1. Yes
   2. No
   3. I am unsure
   4. I do not wish to have children
9. How would you feel about having a child with Fragile X Syndrome?
   1. I would feel more connected to my child.
   2. I would feel conflicted about having a child if I knew he/she would have Fragile X Syndrome.
   3. I would not want to have a child with Fragile X Syndrome.
   4. Not Applicable
   5. Other. Please Explain:
10. Would you opt to use PGD in order to ensure that your child did not have Fragile X Syndrome?

***Preimplantation genetic diagnosis (PGD)*** *is a procedure done before an embryo is implanted (during In Vitro Fertilization or IVF) to help identify genetic conditions within embryos. This serves to prevent certain genetic conditions from being passed on to the child.*

- 1. Yes
  2. I would consider it
  3. No, for mostly moral reasons
  4. No, for mostly economic reasons
  5. I would prefer adoption
  6. Other. Please explain:

1. Please explain any other factors that influence your decisions surrounding reproduction:
